# Histopathology-Based Deep-Learning Predicts Atherosclerotic Lesions in Intravascular Imaging

**DOI:** 10.3389/fcvm.2021.779807

**Published:** 2021-12-14

**Authors:** Olle Holmberg, Tobias Lenz, Valentin Koch, Aseel Alyagoob, Léa Utsch, Andreas Rank, Emina Sabic, Masaru Seguchi, Erion Xhepa, Sebastian Kufner, Salvatore Cassese, Adnan Kastrati, Carsten Marr, Michael Joner, Philipp Nicol

**Affiliations:** ^1^Institute of Computational Biology, German Research Center for Environmental Health, Helmholtz Zentrum München, Oberschleißheim, Germany; ^2^School of Life Sciences Weihenstephan, Technische Universität München, Munich, Germany; ^3^Klinik für Herz- und Kreislauferkrankungen, Deutsches Herzzentrum München, Technische Universität München, Munich, Germany; ^4^Institute of AI for Health, German Research Center for Environmental Health, Helmholtz Zentrum München, Oberschleißheim, Germany; ^5^TUM Department of Informatics, Technische Universität München, Munich, Germany; ^6^Deutsches Zentrum für Herz- und Kreislauf-Forschung (DZHK) e.V. (German Center for Cardiovascular Research), Partner Site Munich Heart Alliance, Munich, Germany

**Keywords:** deep learning, artificial intelligence, intravascular imaging, atherosclerosis, histopathology, optical coherence tomography

## Abstract

**Background:** Optical coherence tomography is a powerful modality to assess atherosclerotic lesions, but detecting lesions in high-resolution OCT is challenging and requires expert knowledge. Deep-learning algorithms can be used to automatically identify atherosclerotic lesions, facilitating identification of patients at risk. We trained a deep-learning algorithm (DeepAD) with co-registered, annotated histopathology to predict atherosclerotic lesions in optical coherence tomography (OCT).

**Methods:** Two datasets were used for training DeepAD: (i) a histopathology data set from 7 autopsy cases with 62 OCT frames and co-registered histopathology for high quality manual annotation and (ii) a clinical data set from 51 patients with 222 OCT frames in which manual annotations were based on clinical expertise only. A U-net based deep convolutional neural network (CNN) ensemble was employed as an atherosclerotic lesion prediction algorithm. Results were analyzed using intersection over union (IOU) for segmentation.

**Results:** DeepAD showed good performance regarding the prediction of atherosclerotic lesions, with a median IOU of 0.68 ± 0.18 for segmentation of atherosclerotic lesions. Detection of calcified lesions yielded an IOU = 0.34. When training the algorithm without histopathology-based annotations, a performance drop of >0.25 IOU was observed. The practical application of DeepAD was evaluated retrospectively in a clinical cohort (*n* = 11 cases), showing high sensitivity as well as specificity and similar performance when compared to manual expert analysis.

**Conclusion:** Automated detection of atherosclerotic lesions in OCT is improved using a histopathology-based deep-learning algorithm, allowing accurate detection in the clinical setting. An automated decision-support tool based on DeepAD could help in risk prediction and guide interventional treatment decisions.

## Introduction

**Figure 1:**
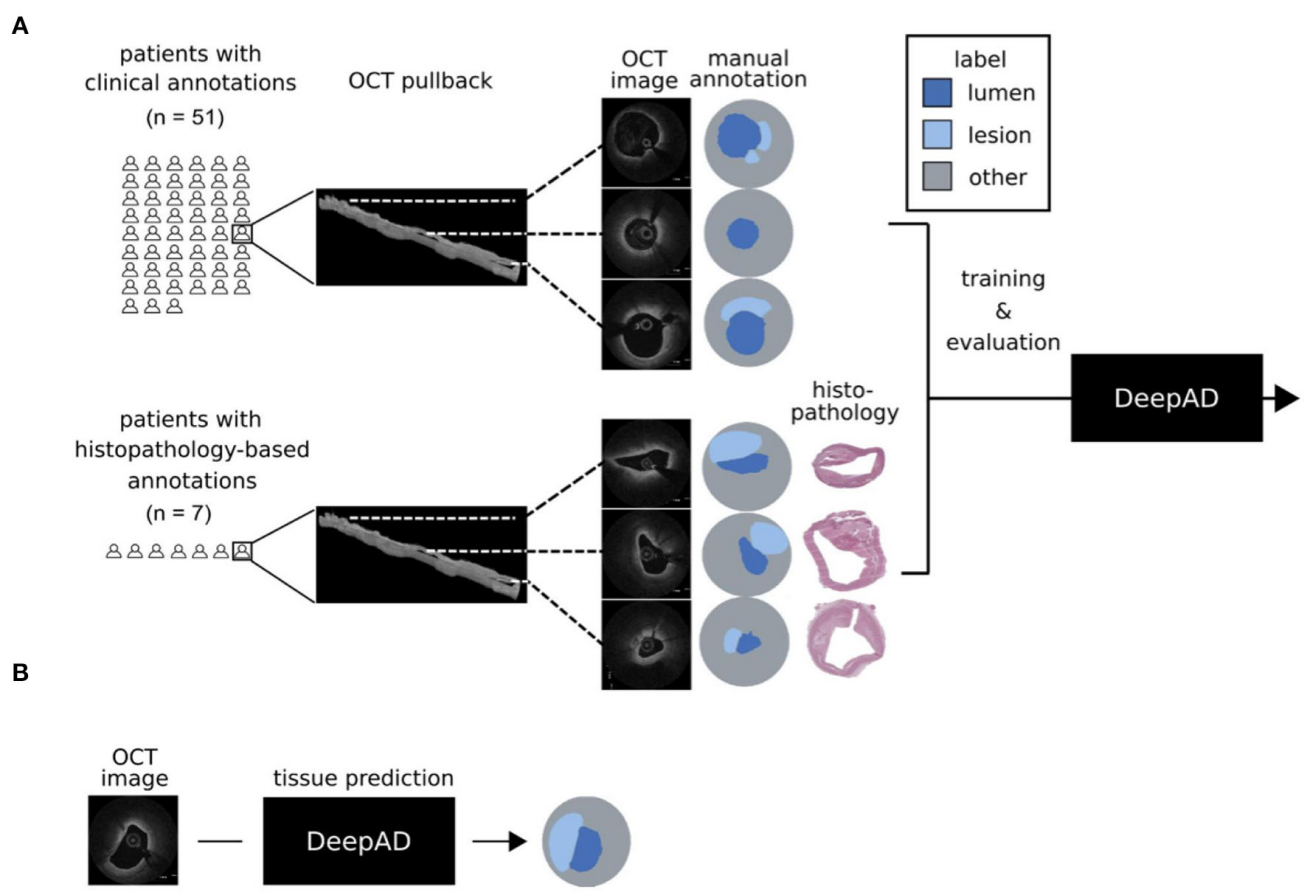
DeepAD for prediction of atherosclerotic lesions from OCT. **(A)** Two data sets were used for training DeepAD: 222 OCT frames from 51 patients with manual annotations based on clinical expertise, and 62 OCT frames from 7 patients with annotations based on co-registered histopathology. **(B)** DeepAD learned to accurately predict atherosclerotic lesions from OCT images and was evaluated on patients with histopathology-based annotations.

Coronary artery disease is the leading cause of death worldwide, accounting for the majority of acute coronary syndromes and sudden cardiac deaths. Identification of atherosclerotic tissue might allow stratification of patients at risk for future coronary events (1–3). Invasive assessment of the coronary arteries using high-resolution intravascular imaging has emerged as an important tool for identifying atherosclerotic lesions (4), as the close proximity of the imaging catheter allows a more precise and high-resolution visualization of the vascular tissue compared to non-invasive modalities (5). The main advantage of optical coherence tomography (OCT) in comparison to other imaging modalities such as intravascular ultrasound (IVUS) is its superb resolution of 10–20 μm with a tissue penetration of approximately 2–3 mm, which enables clear visualization of atherosclerotic components such as lipid tissue with foam cell infiltration or calcifications (5, 6). However, assessment and evaluation of OCT is currently based on clinical expertise from skilled interventionalists and pitfalls in plaque characterization can lead to misclassification (7, 8). Additionally, each volume of OCT images obtained in one measurement, also called a pullback, generates a vast amount of data, precluding from analog assessment of single frames. Due to the high clinical relevance and the cumbersome manual review, automated or semi-automated approaches for preliminary evaluation of OCT images is of great interest for the interventional community (9–11). For intravascular imaging, several groups demonstrated the possibility of automated plaque characterization using semi-automated approaches such as quantification of various optical signal properties (e.g. light attenuation) (12–15). The feasibility of using deep-learning algorithms for detection and visualization of atherosclerotic plaque components was also demonstrated recently (9, 11, 16–19). In this work, we developed DeepAD, a deep-learning algorithm trained on data with histopathology-based annotations from autopsy specimens as well as clinical OCTs for prediction of atherosclerotic lesions, and evaluated it in dedicated real-world cases (Figure 1).

## Methods

### Histopathology and *ex vivo* OCT Imaging

Autopsy samples of coronary arteries were acquired from the department of pathology at Klinikum Rechts der Isar in accordance with federal state law and after approval from relatives (ethical approval number: 291/18 S). Coronary arteries were then cut by an experienced pathologist according to the standard AHA classification. OCT imaging of the single segments was then performed as previously described (15): After careful preparation, vessels were wired with a 0.014-inch guidewire over which the OCT catheter (2.7-F, St. Jude Medical, St. Paul, Minnesota) was advanced. An imaging pullback was performed from the distal to the proximal arterial segments while simultaneously flushing the vessel with contrast to improve imaging quality (pullback speed 5 mm/s = 120 frames/s). Afterwards, arteries were cut at 3 mm intervals and stained with haematoxylin-eosin (H.E.) and Movat-Pentachrome. The presence of different atherosclerotic plaque types and plaque components was evaluated by an expert in cardiovascular pathology (MJ) according to the classification by Virmani et al. (20), (see Table 1). Co-registration of OCT frames and histological sections of human autopsy samples was achieved as previously described (21) by first, considering only the proximal, middle or distal part of the OCT pullback for the respective slide and second, visual alignment of anatomical landmarks (calcifications, side branches, lumen contour) within each part of the segment.

**Table 1:** Histopathological data set (*n* = 62 histopathology images).

**Histopathological** **data set** **N** **=** **62**		**Plaque type**			
		**Pathological intimal thickening (PIT)**	**Fibroatheroma** **(FA)**		**Thin-cap fibroatheroma (TCFA)**
		6/62 (9.7)	45/62 (72.6)		11/62 (17.7)
			**Early FA**	**Late FA**	
			6/45 (13.3)	16/45 (35.6)	
Plaque components	Foam cells	0/6 (0.0)	15/45 (33.3)		8/11 (72.7)
	Calcification	0/6 (0.0)	27/45 (60.0)		4/11 (36.4)
	Necrotic core	0/6 (0.0)	24/45 (53.3)		11/11 (100.0)

*Main characteristics of the histopathological data set regarding presence of different plaque types (PIT, FA and TCFA) and plaque components (Foam cells, calcifications and necrotic core). Values are numbers of frames (percentage)*.

### Clinical Data Set

OCT pullbacks from the OCT database at the German Heart Center Munich (including all patients undergoing intravascular imaging with clinical indication for OCT during coronary angiography) were screened. OCT imaging was performed according to current recommendations (22) with commercially available OCT systems (Dragonfly DF-OCT-catheter combined with the C7-XRTM imaging system; LightLab Imaging Inc., Westford MA, USA). Pullbacks were assessed for the presence of atherosclerotic plaque features as previously described (23) and included fibrous, lipid and calcified plaques (see Table 2). A total of 222 frames from 51 patients were used for analysis. In case of stented vessels, proximal and distal regions outside the stent were used. For baseline characteristics, (see Table 3).

**Table 2:** Clinical data set (*n* = 222 OCT frames).

**Clinical data set** ***N*** **=** **222**		**Plaque type**		
		**Fibrous plaque**	**Lipid plaque**	**Calcified plaque**
		88/222 (39.6)	81/222 (36.5)	53/222 (23.9)
Plaque components	Foam cells	3/88 (3.4)	81/81 (100.0)	28/53 (52.8)
	Calcification	4/88 (4.5)	4/81 (4.9)	53/53 (100.0)
	Lipid pool	2/88 (2.3)	25/81 (30.9)	16/53 (30.2)

*Main characteristics of the clinical data set regarding presence of different plaque types (fibrous plaque, lipid plaque and calcified plaque) and plaque components (Foam cells, calcifications and lipid pool). Values are numbers of frames (percentage)*.

**Table 3:** Baseline characteristics of clinical data set.

*N*		51 (100.0)
Age (years)		66.2 (±14.7)
Gender	Male	35/51 (68.6)
	Female	16/51 (31.4)
CVRF	Arterial hypertension	43/51 (84.3)
	Hypercholesterolemia	42/51 (82.4)
	Diabetes mellitus II	25/51 /49.0)
	Smoker	25/41 (49.0)
CAD	1V-CAD	5/51 (9.8)
	2V-CAD	12/51 (23.5)
	3V-CAD	34/51 (66.7)
Vessel imaged by OCT	LCA	7/51 (13.7)
	LAD	20/51 (39.2)
	LCx	12/51 (23.5)
	RCA	12/51 (23.5)
Indication for OCT imaging	Unclear angiographic findings	2/51 (3.9)
	Guidance of PCI	43/51 (84.3)
	Follow-up after PCI	6/51 (11.8)
Clinical presentation	Silent ischemia	8/51 (15.7)
	Stable AP	31/51 (60.8)
	Unstable AP	4 /51 (7.8)
	ACS	6/51 (11.8)
	Asymptomatic follow-up	2/51 (3.9)

*Values are mean ± SD or n/N (%)*.

### Manual Annotation of OCT Frames

**Figure 2:**
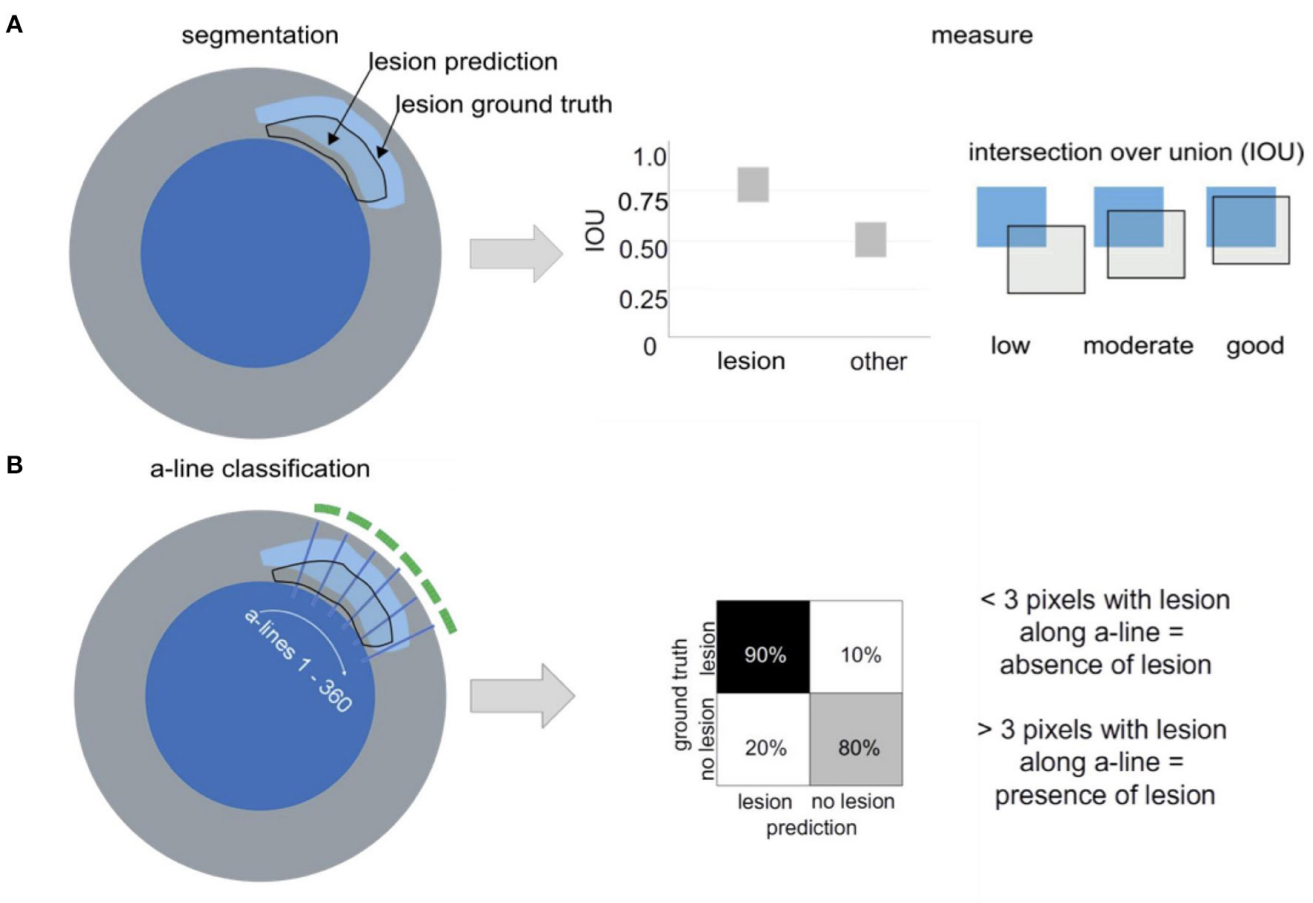
Lesion segmentation vs. a-line classification. **(A)** Lesion segmentation: Atherosclerotic lesions are annotated in OCT frames with or without histopathological information (blue = lumen, gray= vessel wall). Performance of the segmentation approach is evaluated via IOU, measuring the overlap between the labeled “ground truth” and the “prediction” of the algorithm. IOU ranges between 0 and 1 **(B)** a-line classification. Based on the manual annotation, 360 a-line predictions are evaluated per OCT frame regarding the presence or absence of atherosclerotic lesions and can be summarized in a confusion matrix.

OCT images were transferred to an offline working station and transformed into 8bit RGB images. Labeling of OCT frames was achieved using the freeware tool LabelMe (24). In each frame, the suspected plaque area was identified. Plaque area was measured based on histopathology and by tracing the leading edge in OCT images using a polygonal line tool, respectively. Plaque area was defined as the presence of lipid accumulation, necrotic core with or without foam cell infiltration and/or calcification (Figure 2). To avoid pitfalls as reported in (7), we included the guidewire artifact into the label “background”.

### DeepAD

DeepAD is an ensemble of semantic segmentation networks performing pixelwise classification with respect to atherosclerotic lesions. In addition to histopathology-based OCT annotations, we employed so-called weakly supervised training by using OCT images annotated without histopathology. While histopathology-based annotations represent the gold standard, annotations based on OCT images contain valuable information for learning to segment atherosclerotic lesions, however, remain limited in resolution and diagnosis, hence providing a weak supervisory signal to the training (25). We trained and selected several models in so-called ensembles, an effective method to avoid overfitting on small data sets (26). All the models in the ensemble were created with the same network architecture. The differences in the models were the weight initializations of the networks, training batch sampling as well as optimization parameters. As no random seeds were set when training the models, these parameters were assigned different values at the start of each training round. This is a commonly used strategy to achieve different models as the network training of Deep Learning algorithms is stochastic and typically converges to different local minima (27). At inference time, all model predictions were averaged to obtain the final prediction for a given pixel. For further details on network architecture, hyperparameters, exact ensembling, (see Supplementary Material).

### Evaluation on Unseen Patients

To leverage all the available data, DeepAD was trained several times using “leave one patient out” cross-validation. Specifically, for each training round, all histopathology-annotated OCT images from one patient were held out for testing. The remaining data was divided into a 80 % training and 20 % validation split, with no patient overlap between groups. The model was then trained with the training set and tested on the validation set. For each model in the ensemble, the model weights that performed best on the validation set were then tested on the held-out patient. This process was then repeated until each histopathology-annotated patient had been held out for testing once. The OCT images annotated without histopathology were never included in the test set.

### Segmentation

Segmentation describes the task of linking certain regions or pixels within an image to a specific class label (here: lumen, lesion, other). We evaluated the segmentation performance using the intersection over union (IOU, also called Jaccard index), a common metric for evaluation of the quality of segmentation algorithms (28). The IOU is calculated as the overlap between the manual annotation (i.e. the “ground truth”) with the prediction from the algorithm divided by the union (see Figure 2A):


$$\begin{array}{c} \text{IOU}=\text{Area of overlap}/\text{Area of union}. \end{array}$$

IOU can range from 0 (no overlap between ground truth and prediction) to 1 (complete overlap between ground truth and prediction; Figure 2A).

### a-Line Classification

In contrast to segmentation (i.e. the pixel-wise classification), a-line classification computes a binary output regarding the presence of a certain manually annotated class or label in an a-line (e.g. a lesion). In our work, one a-line was defined as one angular direction originating from the center of the lumen. For each OCT frame, 360 a-lines represented the whole 360 ° vessel circumference. Classification was evaluated on a binary level, with a-line being positive if >3 pixels along the a-line contained atherosclerotic lesion and negative if no pixels along the a-line contained atherosclerotic tissue, as defined in previous works (11). Positive a-line signal (indicating presence of atherosclerotic lesion) was visualized by a green line (arch) in the respective vessel circumference. For results of a-line classification, please see Supplementary Material and Supplementary Figure 1.

## Results

### Histopathology and Clinical Data set Characteristics

**Figure 3:**
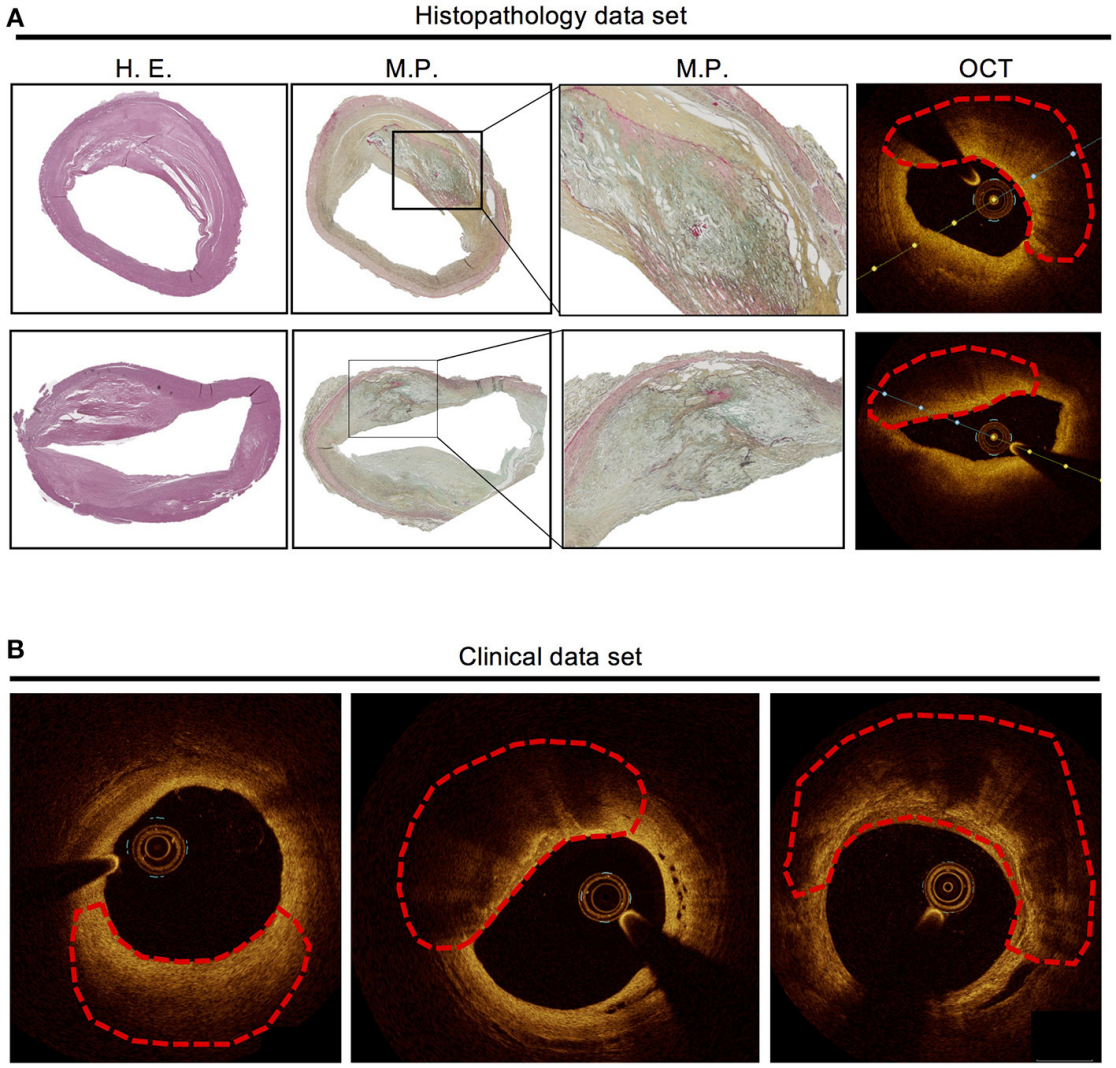
Examples from the histopathology data set and the clinical data set. **(A)** Histopathology of two atherosclerotic lesions (fibroatheroma with necrotic core covered by fibrous cap) with manual annotation (marked by red dashed line) and co-registered OCT frames. **(B)** Three examples of atherosclerotic lesions in clinical OCT images (marked by red dashed line), based on clinical judgement without underlying histopathology. Left: fibrotic plaque, middle: lipid plaque, right: calcified plaque.

In the histopathology data set, a total of 62 lesions from 7 patients were identified according to the classification by Virmani et al. (20) and manually co-registered with OCT (see Table 1 for details regarding the different plaque types) as previously described (21). For the clinical data set, a total of 222 OCT frames were identified as suitable for analysis (see Table 2 for details on the different plaque types). For examples from both datasets (see Figure 3). The histopathology-based annotations as well as the clinical annotations were annotated independently by two clinical and histopathology experts. The estimated interobserver variability for manual annotation of OCT frames with and without histopathology-based annotations was measured as the IOU between two independent expert observers. The median interobserver variability was 0.58 without and 0.71 with histopathology. Thus, the agreement was considerably higher when histopathology-based annotations were available. We also measured the alignment between annotations on the same scans with and without histopathology, being 0.61 across the two annotators. This showed that a difference in results was observed when annotating with or without histopathology.

### DeepAD Accurately Predicts Atherosclerotic Tissue in Unseen Patients

**Figure 4:**
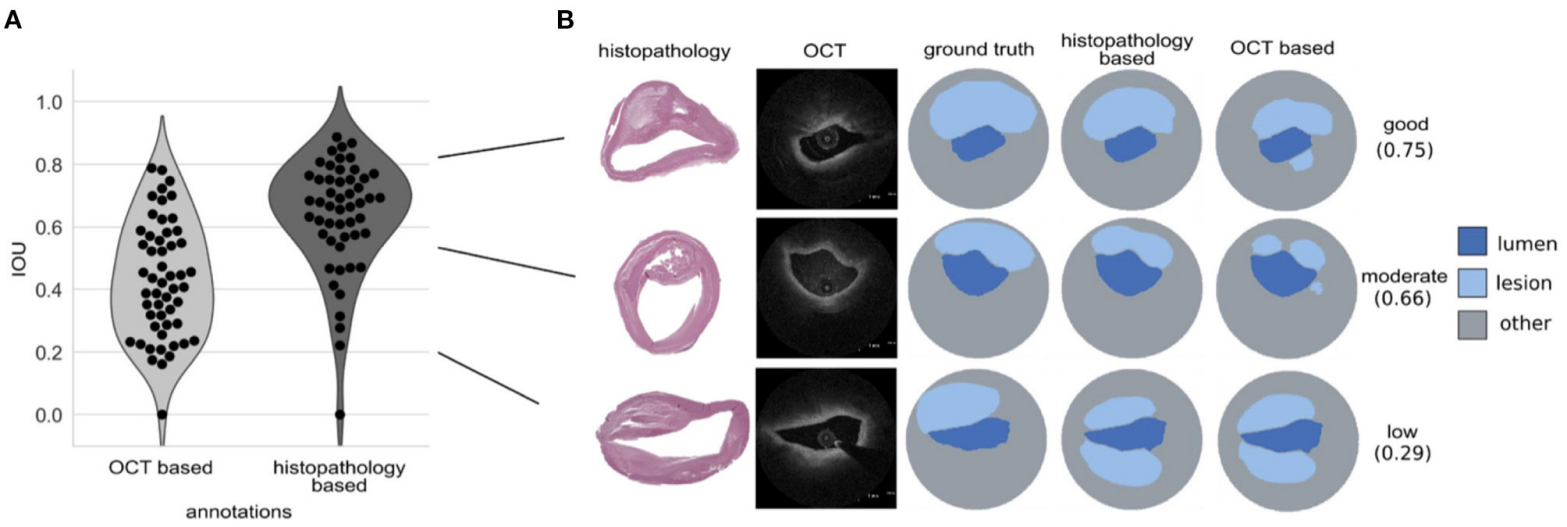
DeepAD predicts atherosclerotic lesion tissue on unseen test patients with good performance. **(A)** Intersection over union (IOU) scores across test sets for atherosclerotic tissue predictions from the OCT only and histopathology-based algorithm (DeepAD), here visualized for every test sample as a dot in the violin plot. **(B)** Examples of predicted atherosclerotic lesions from OCT and histopathology based algorithm: good performance prediction (top, 0.75 IOU, almost all of the region annotated in “ground truth” is predicted by DeepAD), moderate performance (middle, 0.66 IOU, most of the region annotated in “ground truth” is predicted by DeepAD) and low performance (bottom, 0.29 IOU, false-positive prediction of a region from 6-9 o'clock which was not annotated in “ground truth”).

DeepAD predicted atherosclerotic lesions with a median IOU of 0.68 ± 0.18 across all test patients (Figure 4A). Good and moderate performing examples (Figure 4B) illustrate that the DeepADs is highly accurate in the localization of atherosclerotic lesions across the lumen circumference as well as the difficulty of correctly estimating the extent of this lesion beyond the lumen border (see e.g. Figure 4B, moderate example). Figure 4B (lower row) illustrates a low performing prediction from DeepAD. Here DeepAD falsely predicts a lesion below the lumen which is not supported by the manual histopathology-based annotation. Overall, DeepAD predicts lesions with an IOU of 0.03 lower than the median IOU agreement between two observers, showing close to on par segmentations with expert annotators. To evaluate the importance of using histopathology-based OCT annotations, all OCT images in the histopathology data set were additionally annotated without histopathology by two independent observers. The algorithm was then trained with a dataset of the exact same size, but without histopathology-based annotations. The influence and importance of using histopathology-based annotation in the creation of the DeepAD algorithm was measured as the discrepancy of these models' performance with respect to the histopathology-based annotations. When the algorithm was trained using only annotations without histopathology, the median performance on the test set dropped by more than 0.25 IOU from 0.68 ± 0.18 to 0.42 ± 0.18 when predicting atherosclerotic lesion tissue (Figure 4A). This corresponds to a performance drop of around 33 %. In Figure 4B the predictions from DeepADs good, moderate and low performing cases are also shown for the algorithm trained without histopathology. In this case the segmentation algorithm yields false positive predictions (Figure 4B good performing example) and less extensive segmentation beyond the lumen border (Figure 4B median example). In the low performing example in Figure 4B both algorithms output similar false positive tissue prediction.

### Prediction of Calcification by DeepAD

**Figure 5:**
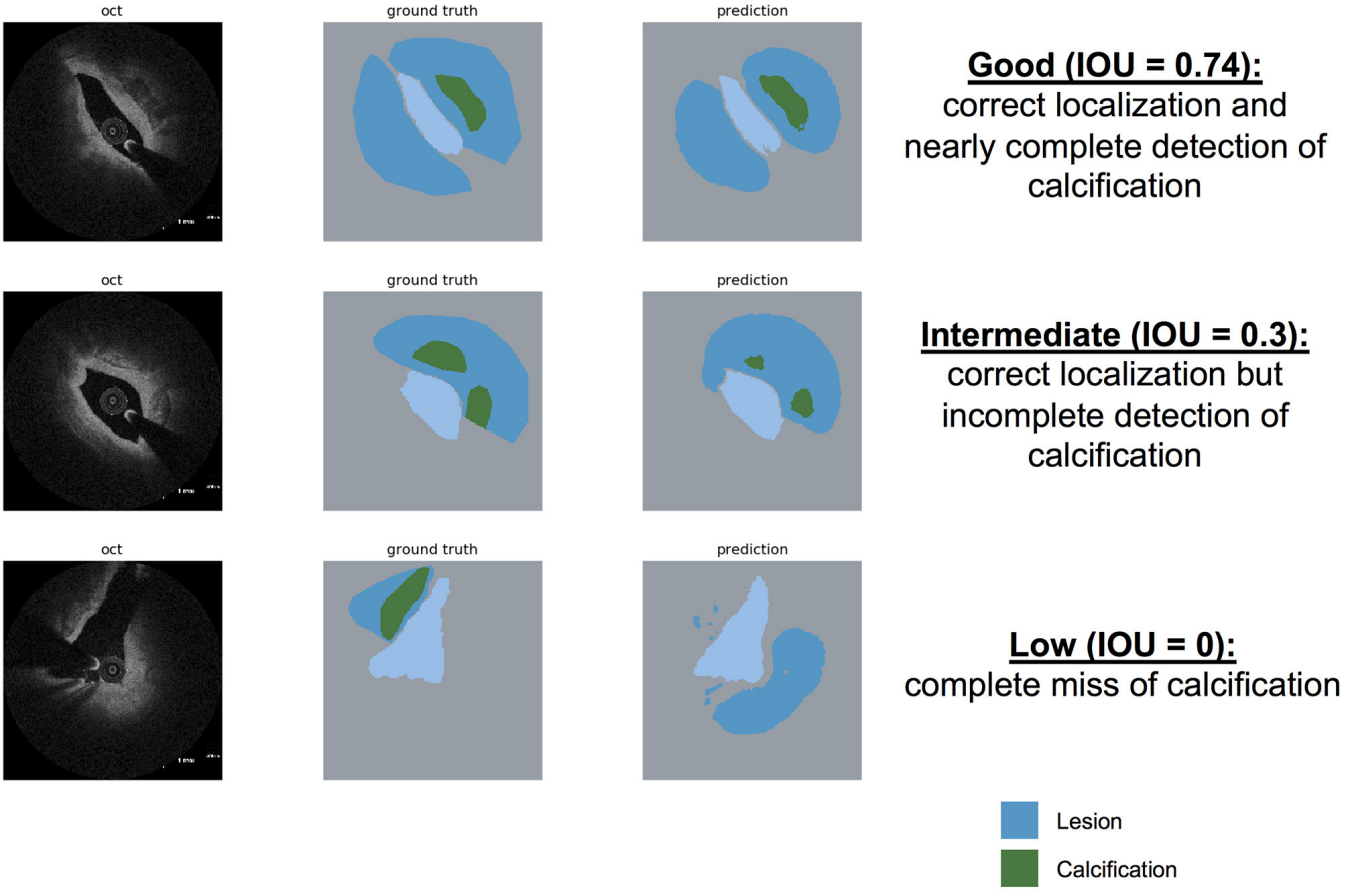
Prediction of calcification by DeepAD. Examples for “good” **(upper row)**, “intermediate” **(middle row)**, and “low” **(lower row)** prediction of calcification using DeepAD (dark blue = lesion, green=calcification, light blue = lumen). Note that with an average IOU = 0.34, most calcification is localized correctly, however with incomplete detection.

Prediction of calcifications in our data set yielded an average IOU = 0.34. Figure 5 shows representative examples of good, intermediate and low performance. In most cases calcification was correctly localized by DeepAD but incompletely predicted (i.e. not all of the calcification was predicted by DeepAD).

### Application of DeepAD in Clinical Cohort

**Figure 6:**
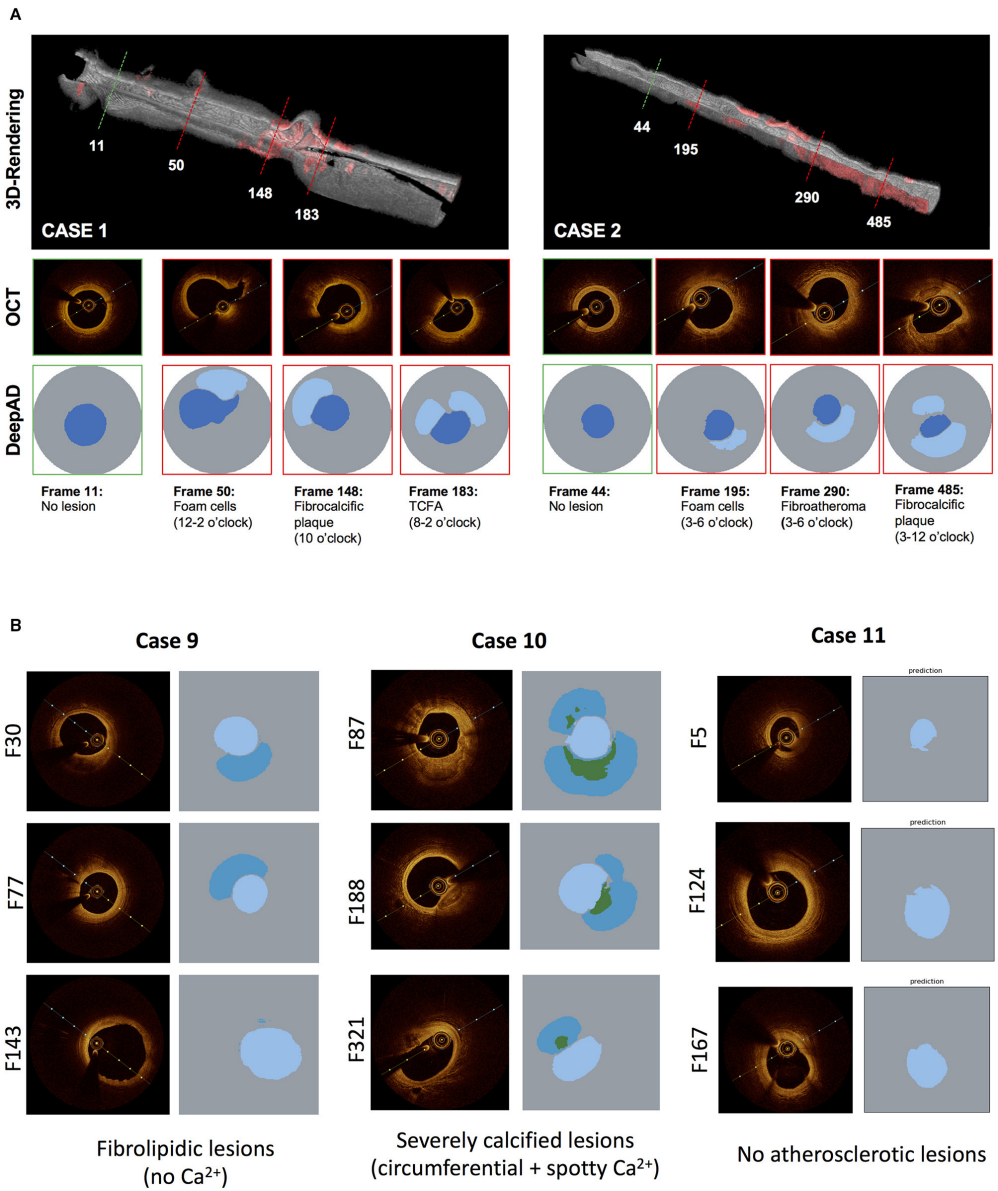
**(A)** Application of DeepAD for lesion detection in clinical cases. 3D reconstruction of OCT pullback visualizes algorithm-based lesions detection (red). Representative cross-sections of healthy (green) and diseased (red lines) areas are shown with respective lesion detection by the DeepAD. **(B)** Subdifferentiation of calcified lesions by DeepAD. Representative OCT frames and corresponding predictions by DeepAD of 3 clinical cases from the clinical cohort (see Table 4). Case 9 mostly has fibrolipidic lesions without any calcifications while case 10 shows heavily calcified lesions (note that DeepAD is able to detect and differentiate circumferential and spotty calcifications). Case 11 shows almost no atherosclerotic lesions.

To illustrate the use of the DeepAD in real-world clinical practice, atherosclerotic lesion prediction was demonstrated in 11 cases of patients who presented to the German Heart Center and underwent coronary angiography and intravascular imaging with OCT. Performance of the algorithm was retrospectively compared against manual analysis of each pullback by an expert regarding the presence or absence of atherosclerotic lesions as well as correct spatial prediction of the DeepAD on a frame-level. 3D-rendering of the complete pullback allowed quick and intuitive visualization of regions with atherosclerotic lesions in red, (see Figure 6A). A total of 3284 frames were analyzed and high agreement was seen between automated prediction by DeepAD and manual analysis (mean agreement 88%, 2898 of 3284 frames), with good sensitivity, specificity and accuracy (86.8, 82.9 and 85.8%, see Table 4). DeepAD tended to slightly underestimate the presence of atherosclerotic lesions (66 vs. 71% by manual analysis). Representative frames of false-positive predictions are shown in Supplementary Figure 2. For examples on detection of calcification, (see Figure 6B).

**Table 4:** Application of DeepAD in clinical cohort (n=11 cases) and comparison with manual analysis.

**Case**	**No lesion** **% (n/N frames)**		**Lesion present** **% (n/N frames)**		**Agreement % (n/N frames)**	**Sensitivity %**	**Specificity %**	**Accuracy %**
	**DeepAD**	**Manual analysis**	**DeepAD**	**Manual analysis**				
1	60 (165/275)	68 (187/275)	40 (110/275)	32 (88/275)	88 (241/275)	90.9	83.9	86.2
2	44 (239/538)	38 (206/538)	56 (299/538)	62 (332/538)	93 (501/538)	88.0	96.6	91.3
3	20 (24/118)	5 (6/118)	80 (94/118)	95 (112/118)	83 (98/118)	83.0	83.3	83.1
4	1 (7/477)	0 (0/477)	99 (470/477)	100 (477/477)	91 (470/477)	98.5	n.a.	98.5
5	52 (135/262)	42 (110/262)	48 (127/262)	58 (152/262)	69 (181/262)	65.1	74.5	69.1
6	20 (48/243)	9 (22/243)	80 (195/243)	91 (221/243)	86 (209/243)	86.4	81.8	86.0
7	46 (156/338)	46 (154/338)	54 (182/338)	54 (184/338)	93 (314/338)	92.9	92.9	92.9
8	18 (37/202)	20 (40/202)	82 (165/202)	80 (162/202)	89 (179/202)	93.8	67.5	88.6
9	18 (28/156)	8 (12/156)	82 (128/156)	92 (144/156)	87 (136/156)	87.5	83.3	87.2
10	21 (88/424)	0 (0/424)	79 (336/242)	100 (424/424)	79 (336/424)	79.2	n.a.	79.2
11	75 (189/251)	87 (228/251)	25 (62/251)	13 (23/251)	82 (206/251)	87.0	81.6	82.1
Total/ mean	34 (1116/3284)	29 (965/3284)	66 (2168/3284)	71 (2319/3248)	88 (2898/3284)	86.6 (±8.9)	82.9 (±8.7)	85.8 (±7.7)

## Discussion

The goal of our study was the development of a fully-automated, deep-learning algorithm trained on histopathology and clinical data for the prediction of atherosclerotic lesions in intravascular OCT. The main findings of our work are:

I DeepAD is able to predict atherosclerotic lesions in intravascular OCT images when trained on data from histopathology and clinical imaging, with an IOU = 0.68 (±0.18).
II Although prediction of calcification was lower (IOU = 0.34), calcified lesions were localized correctly in most cases (circumferential and spotty).
III Using histopathology-based annotations helped in training the algorithm over using only clinical annotations.
IV Clinical applicability of DeepAD was demonstrated in a small clinical cohort of 11 cases.

Application of deep-learning technology has the ability to transform the biomedical field in the 21th century. Due to significant advancements in access and storage of big data, and the availability of adequate processing power, use of algorithms embedded in artificial intelligence is likely to have a considerable impact on clinical practice toward more standardized and personalized patient care (29). The need for promoting artificial intelligence in the field of intravascular imaging is supported by the fact that the consequenes of advanced and undetected coronary artery disease are still detrimental, with cardiovascular disease being the leading cause of death worldwide and acute coronary syndromes or stroke accounting for 85% of these deaths (30). This hypothesis is supported by two large-scale studies: using virtual-histopathology IVUS (VH-IVUS) in 697 patients presenting with ACS, Stone et al. showed that lesions characterized as thin-cap fibroatheroma, although being angiographically mild, were responsible for the majority of non-culprit coronary events after 3 years (4). Similar, Prati et al. found that the presence of four high-risk plaque features identified by OCT (minimal lumen area <3.5 mm_2_ fibrous cap thickness <75 mm, lipid arc circumferential ex- tension >180° and infiltration with macrophages) was associated with a higher risk of coronary events in 1,003 patients (31). Although all clinically used intracoronary imaging modalities (IVUS, OCT and NIRS) possess inherent limitations, the availability and application of high-resolution intracoronary imaging will likely increase with time (8). Most recently, NIRS (using near-infrared spectroscopy to visualize lipid-rich plaques) has been shown to detect patients at a higher risk for subsequent coronary events (32), being the first intracoronary imaging modality with the ability to provide predictive assessment of atherosclerotic coronary lesions. In our work, DeepAD predicted atherosclerotic lesions in general with high accuracy. While the identification of vulnerable plaques might be desirable and could be regarded as the “holy grail” in cardiovascular imaging, it may be argued that the concept of a single “vulnerable” plaque leading to coronary events might be overly simplistic and that rather the patient himself should be considered vulnerable. In this regard, detection of atherosclerosis burden might help in stratification of patients as the presence of atherosclerosis correlates with cardiovascular events (33, 34). For example, prospective studies have shown that identification of coronary artery calcification can be used for risk prediction in patients with suspected coronary artery disease and is therefore utilized in clinical risk scores (35). This seems contradictory at first as it is known from studies on vascular biology that calcification per se is an important mechanism of plaque stabilization: while macrophages are undergoing apoptosis, microcalcifications are occurring as a consequence of cell death (36). Eventually, the whole necrotic core might calcify over time, leading to stabilization and passivation of high-risk plaque regions (36). Nevertheless, calcification is closely correlating with overall atherosclerotic burden and therefore predicts mortality in patients with suspected coronary artery disease (37). Hence, similarities to our approach of using deep learning for detection of atherosclerotic burden with OCT are obvious: DeepAD enables identification of coronary atherosclerosis, which can be visualized using OCT but often pose a challenge in daily clinical practice for inexperienced clinician.

When detecting calcification, performance of DeepAD yielded an IOU = 0.34 (±0.07). While this is definitely inferior to the detection of atherosclerotic lesions in general (IOU = 0.68), we demonstrate that in most cases, calcification is correctly localized by DeepAD but incompletely predicted (i.e. not the complete extent of the calcification is predicted by DeepAD). For the interventionalist, this limitation might be neglectable as the sheer presence of calcification (even though not completely detected) is highly informative. This in turn might influence interventional treatment strategy (use of high-pressure or cutting-balloon, pre- or post-dilatation etc.) to deliver an optimal procedural result and avoid e.g. incomplete stent expansion.

With respect to previous work, only one study used co-registered histopathology images with OCT images for plaque segmentation (16). While our work is relying on a deep-learning approach to segment atherosclerotic lesions, the work by He at al. used *ex vivo* carotid plaque tissue samples and different, carefully handcrafted features with a decision tree. In our approach, CNN learns all filters automatically without any human supervision, hence a decision tree is not needed this way. Besides, deep-learning methods that do not use histopathology images have a limitation on the annotation quality: When segmenting without the help of histology images, only features which are clearly visible in the OCT image will be annotated and tissue that reaches further out will probably be missed. With the help of histopathology, these regions can be annotated more accurately. This ideally enables DeepAD to predict even hard-to-detect features that likely would have been missed if trained on clinical OCTs only.

Ultimately, DeepAD should be regarded as a decision-supporting tool which can aid in the clinical setting by providing a more standardized characterization of OCT with the potential to quickly visualize atherosclerotic burden in the cath lab. Whether this may be used for risk prediction has to be validated in dedicated future studies. In conclusion, our work highlights the value of using artificial intelligence in the field of intracoronary imaging. By providing an easily applicable tool based on a deep-learning algorithm trained with histopathology-annotated data as well as with clinical expertise, DeepAD can ultimately improve the diagnostic performance of intracoronary optical coherence tomography in detecting atherosclerotic lesions.

## Data Availability Statement

The raw data supporting the conclusions of this article will be made available by the authors, upon reasonable request.

## Ethics Statement

The studies involving human participants were reviewed and approved by Ethical Committee of TU München, Ethical Approval Number: 291/18 S. The patients/participants provided their written informed consent to participate in this study.

## Author Contributions

All authors listed have made a substantial, direct, and intellectual contribution to the work and approved it for publication.

## Funding

This work was supported through a scientific grant by the German Cardiac Society (Grant Number: 16/2020). CM has received funding from the European Research Council (ERC) under the European Union's Horizon 2020 research and innovation programme (Grant agreement No. 866411).

## Conflict of Interest

MJ reports personal fees from Biotronik, personal fees from Orbus Neich, grants and personal fees from Boston Scientific, grants and personal fees from Edwards, personal fees from Recor, personal fees from Astra Zeneca, grants from Amgen, personal fees from Abbott, personal fees from Shockwave, grants from Infraredx, grants from Cardiac Dimensions outside the submitted work. The remaining authors declare that the research was conducted in the absence of any commercial or financial relationships that could be construed as a potential conflict of interest.

## Publisher's Note

All claims expressed in this article are solely those of the authors and do not necessarily represent those of their affiliated organizations, or those of the publisher, the editors and the reviewers. Any product that may be evaluated in this article, or claim that may be made by its manufacturer, is not guaranteed or endorsed by the publisher.

## Abbreviations

CAD: Coronary artery disease
CNN: Convolutional neural network
CVRF: Cardiovascular risk factors
DES: drug-eluting stent
H.E.: Hematoxylin and eosin
IDDM: insulin-dependent diabetes mellitus
IOU: intersection over union
IVUS: intravascular ultrasound
LAD: Left anterior descending artery
LCA: Left coronary artery
LCX: Left circumflex artery
M.P.: Movat-pentachrome
NIRS: near-infrared spectroscopy
OCT: Optical coherence tomography
PCI: Percutaneous coronary intervention
RCA: Right coronary artery.
