# Supplementary material for: Histopathology-Based Deep-Learning Predicts Atherosclerotic Lesions in Intravascular Imaging
Source: Front Cardiovasc Med. 2021 Dec 14;8:779807. doi: 10.3389/fcvm.2021.779807 (PMC8713728; doi:10.3389/fcvm.2021.779807)
Supplement: Supplementary file 1 [file Data_Sheet_1.docx]

**Supplement**

**Supplemental methods**

**Exact model architecture of DeepAD convolutional neural networks**

Below a detailed view of the exact model architecture of the DeepAD CNN models is given. First, the input image is resized to 256 by 256 pixels. The network then consists of a total of 28 convolutional layers configured with the following number of filters. The first convolutional layer is initialized with 64 filters. This number is then doubled four times, each after two convolutional layers have been applied, with the exception for the fourth doubling, at 512 filters where 6 convolutional layers are applied. This is done until 1024 filters are operating in the so-called bottleneck of the encoder decoder architecture. Between every convolutional layer batch normalization [(1)](https://paperpile.com/c/NuoNsv/BfQz) and a ReLu [(2)](https://paperpile.com/c/NuoNsv/Q5cA) activation function is applied and after every two convolutional layers MaxPooling is applied halving the dimension of the feature maps ending at a dimension of 8x8 in the bottleneck of the network. Each convolution is performed using padding to leave the feature map unchanged and uses a stride of 1 in both width and height direction. After the first 12 convolutional layers with batch normalization and ReLu activation functions applied the decoder network starts. The decoder network consists of 16 convolutional layers of which 5 are convolutional transpose layers upscaling the features back to size 256x256. Also, in the decoder each convolutional layer is followed by batch normalization as well as a ReLu activation function. The final convolutional layer is the final softmax layer, yielding the probabilities for all classes and pixels. As in the U-net [(2)](https://paperpile.com/c/NuoNsv/E2Qq) architecture intermediate layers in the encoder are concatenated to their dimensional counterparts in the decoder, in this network a total of five such concatenations are performed. In total the network architecture used has 37 899 279 trainable parameters.

**Model Ensembling and hyperparameter information**

For the training of the DeepAD model ensemble, five deep convolutional neural networks (CNN) with a U-net architecture were used for segmenting the OCT images (2, 3). After each convolutional layer a batch normalization layer was used (4). Each model was trained independently on both data sets (Fig. 1 a). The models were trained using cross entropy optimized by the Adam optimizer [(5)](https://paperpile.com/c/t6gBTa/Hhprd) with an initial learning rate of 0.001, yielding the highest performance after hyperparameter optimization. Standard data augmentations such as rotation, flipping and brightness distortions were randomly applied to the training data during training but not during testing. For rotation, an image was rotated with a probability of 0.5 by a uniformly sampled angle between 0 and 90. Both horizontal and vertical flipping was performed with a probability of 0.5 and finally brightness distortions were applied with a probability of 0.5 with a limit of ∓0.6. At inference time, each network in the ensemble independently segments an OCT image and for the final prediction the class receiving the highest average prediction score across the five models was selected for each pixel.

**Inference time of DeepAD**

An important aspect of the DeepAD algorithm is its inference time, predicting atherosclerotic tissue in OCT images. While the inference time of DeepAD depends on which hardware is used to perform inference, in this work, we used a Nvidia GTX 1080 GPU and measured the inference time of one image at a time. The inference time of DeepAD measures to about 200 milliseconds per image. For a representative OCT pullback containing 300 frames this would amount to 60 seconds inference time for the entire OCT pullback. It is important to note that this inference time could be increased or also decreased through the change of hardware and batch inference.

**DeepAD tissue prediction evaluated by a-line classification accuracy**

The prediction performance of DeepAD can be also assessed using a-line analysis. In supplementary figure 2, the same examples from Fig. 4a are presented with regards to a-line classification. Notably, the moderate-performing example from Figure. 4a, where atherosclerotic tissue is less segmented beyond the lumen, has a seemingly smaller effect on the performance in a-line classification. Overall, DeepAD accurately detects the presence or absence of atherosclerotic lesions with 88 % and 89 % accuracy, respectively, summed over all histopathology annotated cases (Suppl. Fig. 2b).

**References**

1. [Ioffe S, Szegedy C. Batch Normalization: Accelerating Deep Network Training by Reducing Internal Covariate Shift. In: Bach F, Blei D, editors. Proceedings of the 32nd International Conference on Machine Learning. Lille, France: PMLR; 2015. p. 448–56. (Proceedings of Machine Learning Research; vol. 37).](http://paperpile.com/b/t6gBTa/7mxGc)

2. [LeCun Y, Bengio Y, Others. Convolutional networks for images, speech, and time series. The handbook of brain theory and neural networks. 1995;3361(10):1995.](http://paperpile.com/b/t6gBTa/9Jm1Q)

3. [Ronneberger O, Fischer P, Brox T. U-Net: Convolutional Networks for Biomedical Image Segmentation. In: Medical Image Computing and Computer-Assisted Intervention – MICCAI 2015. Springer International Publishing; 2015. p. 234–41.](http://paperpile.com/b/t6gBTa/MB009)

4. [Lakshminarayanan B, Pritzel A, Blundell C. Simple and Scalable Predictive Uncertainty Estimation using Deep Ensembles [Internet]. arXiv [stat.ML]. 2016. Available from:](http://paperpile.com/b/t6gBTa/B9Oya) <http://arxiv.org/abs/1612.01474>

5. [Kingma DP, Ba J. Adam: A Method for Stochastic Optimization [Internet]. arXiv [cs.LG]. 2014. Available from:](http://paperpile.com/b/t6gBTa/Hhprd) <http://arxiv.org/abs/1412.6980>

**Supplementary Figures**

**Supplementary Figure 1: Atherosclerotic tissue detection by DeepAD using a-line classification.**

*(a) a-line classification visualized as green arches for examples from Figure 4.*

** “performance” refers to the IOU performance from the examples listed in figure 4. Here the good performing example has 99 %, moderate example 97 % and low performing example 56 % a-line accuracy. Notably the moderate performing example generates a high a-line accuracy as the failure to segment lesions far beyond the lumen does not inhibit the detection of lesions in this part of the OCT. (b), Confusion matrix showing binary classification of DeepAD regarding the presence or absence of atherosclerotic lesions with an accuracy of 88 % and 89%, respectively, see panel b.*

**Supplementary Figure 2: Examples of false prediction by DeepAD in clinical case 1**
