# Supplementary figures and images for: Histopathology-Based Deep-Learning Predicts Atherosclerotic Lesions in Intravascular Imaging
Source: Front Cardiovasc Med. 2021 Dec 14;8:779807. doi: 10.3389/fcvm.2021.779807 (PMC8713728; doi:10.3389/fcvm.2021.779807)

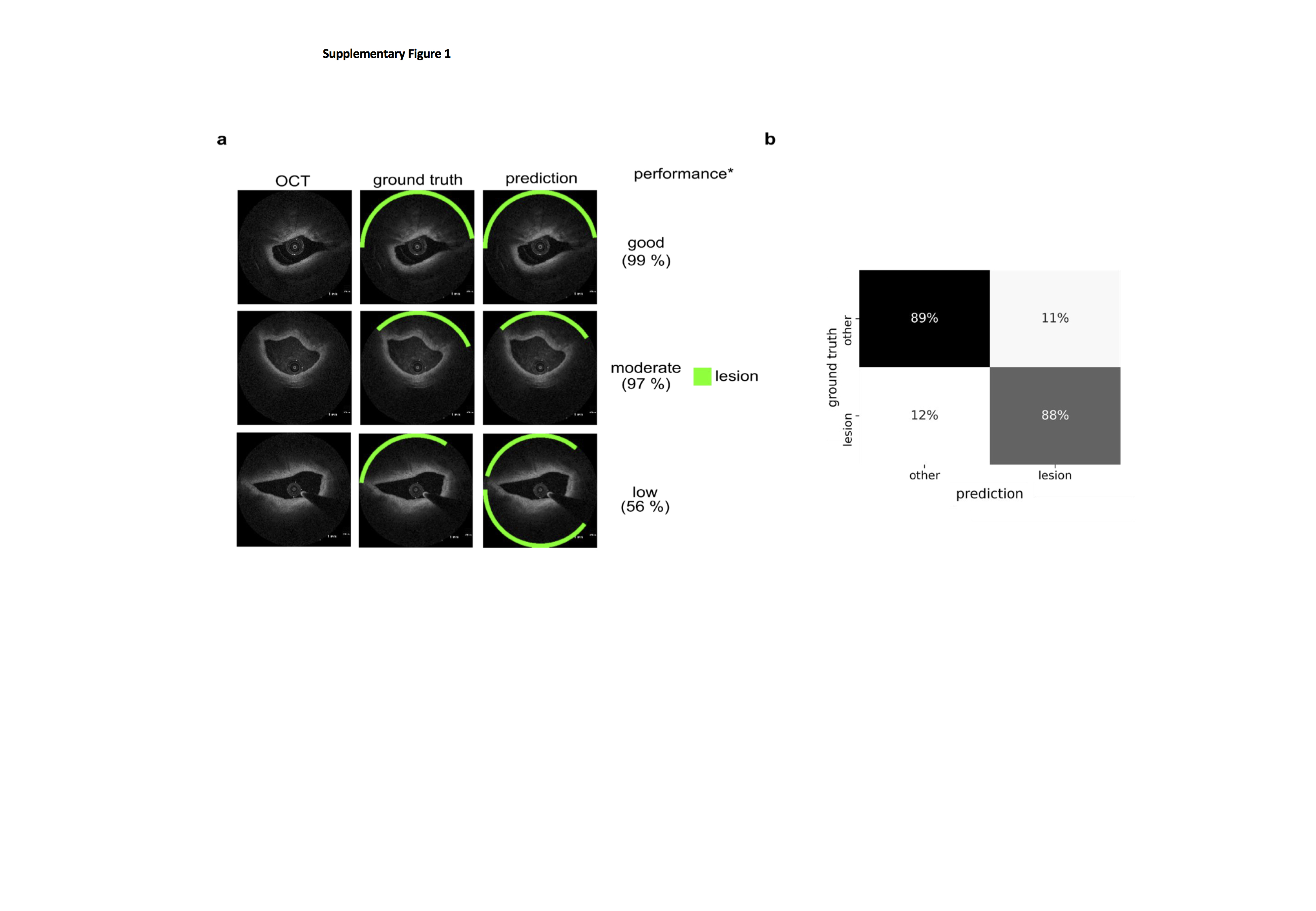

Supplement: Supplementary file 2 [file Image_1.jpg]

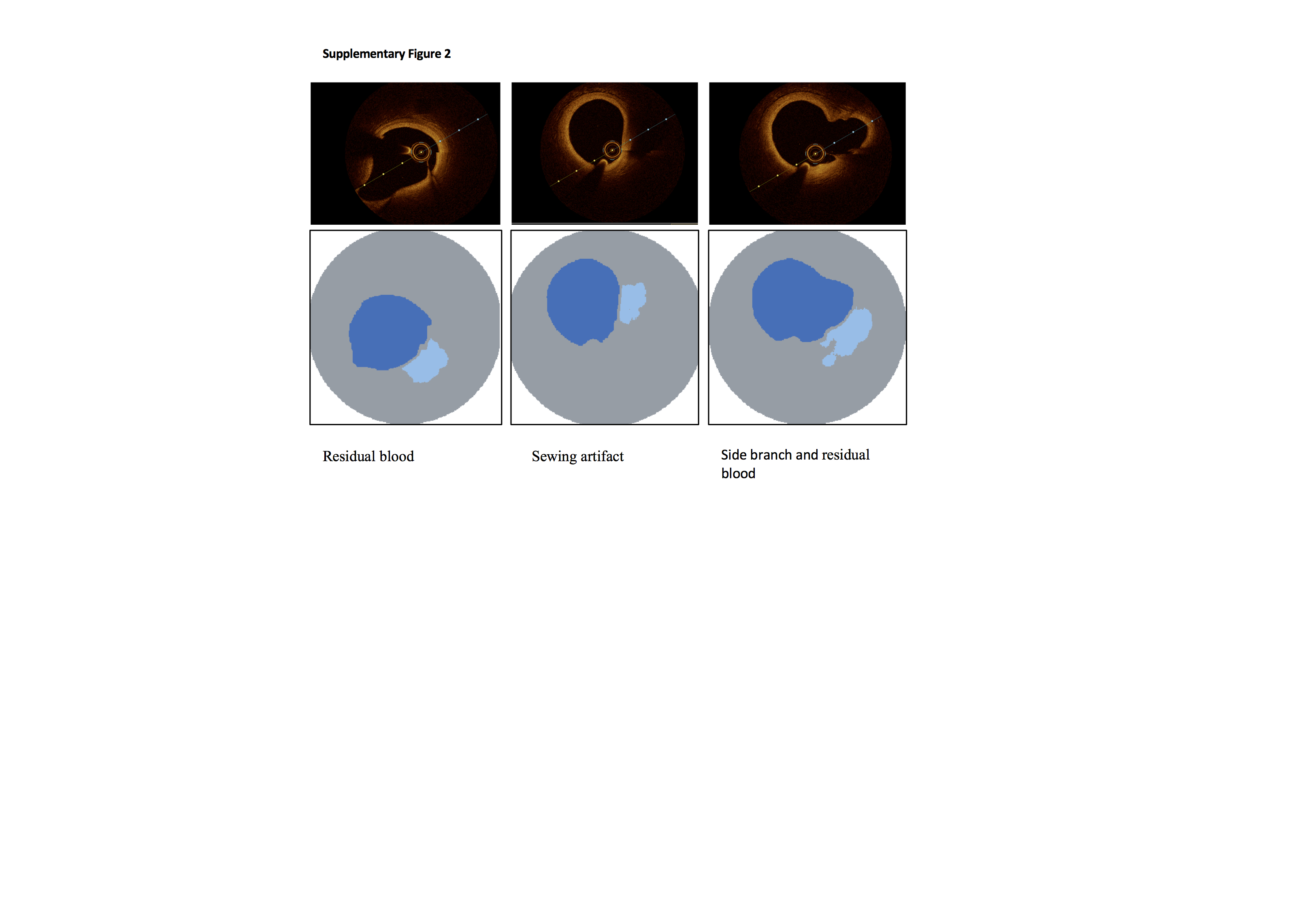

Supplement: Supplementary file 3 [file Image_2.jpg]
